# Investigating reindeer pastoralism and exploitation of high mountain zones in northern Mongolia through ice patch archaeology

**DOI:** 10.1371/journal.pone.0224741

**Published:** 2019-11-20

**Authors:** William Taylor, Julia K. Clark, Björn Reichhardt, Gregory W. L. Hodgins, Jamsranjav Bayarsaikhan, Oyundelger Batchuluun, Jocelyn Whitworth, Myagmar Nansalmaa, Craig M. Lee, E. James Dixon

**Affiliations:** 1 Department of Archaeology, Max Planck Institute for the Science of Human History, Jena, Germany; 2 Museum of Natural History, University of Colorado-Boulder, Boulder, Colorado, United States of America; 3 Department of Archaeology, Flinders University, Adelaide, Australia; 4 NOMAD Science, Ulaanbaatar, Mongolia; 5 Central Asian Seminar, Institute for Asian and African Studies, Humboldt-Universität zu Berlin, Berlin, Germany; 6 Department of Archaeogenetics, Max Planck Institute for the Science of Human History, Jena, Germany; 7 Accelerator Mass Spectrometry Laboratory, University of Arizona, Tucson, Arizona, United States of America; 8 National Museum of Mongolia, Ulaanbaatar, Mongolia; 9 Clearview Animal Hospital, Colorado Springs, Colorado, United States of America; 10 Unit of Diagnosis and Surveillance for Infectious and Parasitic Disease, State Central Veterinary Laboratory, Ulaanbaatar, Mongolia; 11 Institute for Arctic and Alpine Research, University of Colorado-Boulder, Boulder, Colorado, United States of America; 12 Department of Anthropology, University of New Mexico, Albuquerque, New Mexico, United States of America; Washington University in Saint Louis, UNITED STATES

## Abstract

In interior Eurasia, high mountain zones are crucial to pastoral subsistence, providing seasonally productive pastures and abundant wild resources. In some areas of northern Mongolia, mountainous tundra zones also support a low-latitude population of domestic reindeer herders–a lifestyle whose origins are poorly characterized in the archaeological record of early Mongolia. Traditionally, reindeer pastoralists make significant seasonal use of *munkh mus* (eternal ice) for their domestic herds, using these features to cool heat-stressed animals and provide respite from insect harassment. In recent years, many of these features have begun to melt entirely for the first time, producing urgent threats to traditional management techniques, the viability of summer pastures, and reindeer health. The melting ice is also exposing fragile organic archaeological materials that had previously been contained in the patch. We present the results of horseback survey of ice patches in Baruun Taiga special protected area, providing the first archaeological insights from the region. Results reveal new evidence of historic tool production and wild resource use for fishing or other activities, and indicate that ice patches are likely to contain one of the few material records of premodern domestic reindeer use in Mongolia and lower Central Asia. The area’s ancient ice appears to be rapidly melting due to changing climate and warming summer temperatures, putting both cultural heritage and traditional reindeer herding at extreme risk in the years to come.

## Introduction

In the cold, dry, and harsh environs of the Eurasian steppes, mountain regions have played a key role in herding lifeways both now and in the past -while contemporary climate warming threatens both pastoral adaptations and the region’s fragile archaeological record. In contemporary Mongolia, most rural residents make their living through specialized, seasonally-mobile herding of domestic livestock. Across most of the region, the key herding animals are known the *tavan hoshuu mal* (five snouts)–sheep (*Ovis aries*), goat (*Capra hircus*), horse (*Equus caballus*), cattle (*Bos taurus*), and camel (*Camelus bactrianus)*. Montane environments in the dry steppe function as important loci of animal and plant biodiversity for hunting, fishing, and gathering, as well as focal points for diverse kinds of pastoral activity. In dryland regions of Central Asia, precipitation is scarce and highly seasonal; orographic precipitation generated by mountain topography is crucial to pasture growth (Frachetti 2008). Improved rain and pasture reliability of montane zones enables herders to practice vertical seasonal movements over short geographic distances into alpine summer pastures, where the well-watered forage can sustain larger populations of heavier, more water dependent livestock, as well as the cold-adapted yak (*Bos grunniens*) and the reindeer (*Rangifer tarandus*), which are found only in northern Mongolia. Owing to these unique ecological roles, montane regions in Mongolia appear to have been hotspots for the region’s earliest herding economies during the early Bronze Age (Taylor et al in review).

In recent years, consideration of paleoenvironmental proxies alongside archaeological data has revealed close links between climate processes important pastoral social developments in northeast Asia. For example, changes in temperature and precipitation regimes have been linked to the initial transition to herding from hunting and gathering [1], the adoption of domestic horses [2], and the formation of nomadic empires [3]. In the modern era, Mongolia is witnessing climate warming at rates exceeding the global average, with summer temperatures already 1.5°C warmer than 20th century levels as of 2001 [4].As climate changes continues to produce extreme warming across Northeast Asia, characterizing the relationship between climate processes and herding life both now and in antiquity is fundamental to understanding the future of pastoral economies that characterize a wide geographic region.

**Fig 1:**
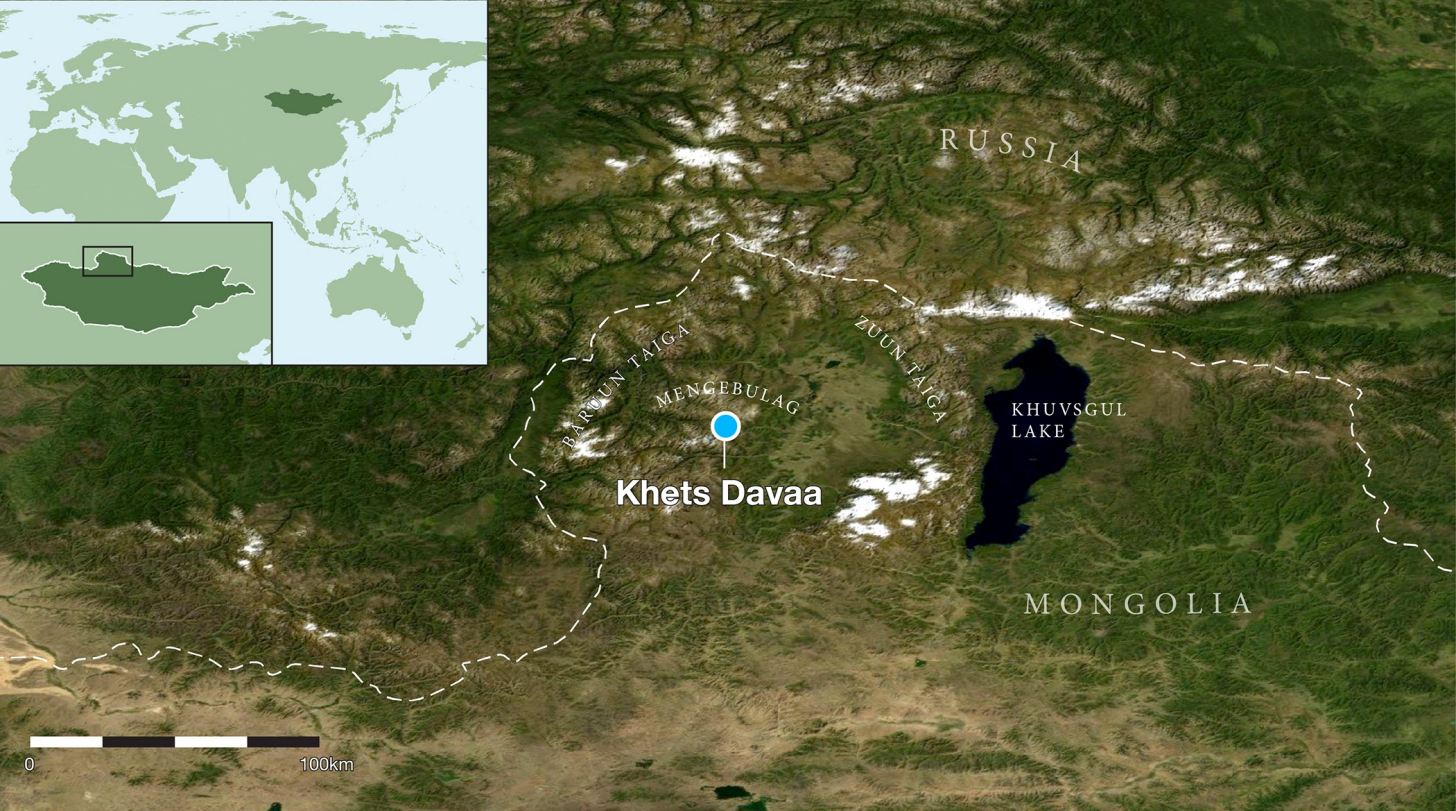
Map showing the sites discussed in the text, with the location of key political and geographic reference points. We acknowledge the use of imagery provided by services from NASA's Global Imagery Browse Services (GIBS), part of NASA's Earth Observing System Data and Information System (EOSDIS).

High altitude tundra zones found in northern Mongolia’s Sayan Mountains (Fig 1) are particularly threatened by climate warming. Although the early history and prehistory of pastoralism in this region is poorly understood, several lines of evidence suggest that the area could yield crucial insights into the premodern history of domestic reindeer herding. Along the Russian border in Khuvsgul province, lichen-rich tundra and larch forest mountain zones, historically home to wild reindeer, are interdigitated with areas of pasture that have been home to specialized livestock herders for more than 3000 years [5]. Today, the area is the world’s lowest-latitude location (~51° N) where reindeer pastoralism is practiced. Consequently, some scholars have highlighted the region as a promising location for the animal’s initial domestication [6,7].Unfortunately, despite a rich archaeological record in the grasslands of the nearby Darkhad area, years of survey and anthropological fieldwork have yielded almost no artifacts or cultural material from the mountain tundra. This scenario leaves researchers with little basis for understanding the prehistory of reindeer pastoralism northern Mongolia, or its relationship to processes of climate and environmental change.

Perennial deposits of snow and ice, known as ice patches, provide a promising avenue for answering these critical questions. These non-glacial accumulations often occur on north-facing slopes in high latitude and high altitude regions. Ice patches are distinguished by the fact that they do not melt completely during the summer months. Because of their perennial nature, ice patches play a crucial role in summer subsistence for many plant and animal taxa, providing thermal relief for wild reindeer [8], reprieve from insects [9], and a source of reliable freshwater and useful plant species [10] for herder and herded alike. In areas where wild reindeer and caribou are abundant, ice patches have served as predictable reindeer hunting grounds around the world in prehistory, e.g. [11–13] and historic times [14]. The stable, frozen environment presented by ice patches preserves organic artifacts which enter the ice, offering an opportunity to explore a unique material record of high altitude subsistence activities–particularly those related to reindeer. Archaeological finds from arctic and subarctic regions in globally, such as Alaska and Scandinavia, include ‘scare sticks’ used in reindeer drives, projectiles, skis, baskets used to collect berries or snow, and even culturally modified reindeer hide fragments [12, 15,16]. In cases where non-glacial, perennial ice has remained stable in areas of cultural activity, ice patches have preserved artifacts dating back more than 10,000 years [17]. For these reasons, ice patches in northern Mongolia are likely to retain a signature of premodern subsistence activities at high altitude which might provide new and unique insights into the history of domestic reindeer in Northeast Asia’s lower latitudes.

### Study area

Nestled alongside the large Darkhad Depression (Fig 1) to the west of Khuvsgul Lake along the Siberian border, the Ulaan Taiga Special Protected Area is an expansive area of tundra and larch forest, home to the reindeer-herding Tsaatan people(ethnic Tuvan or *Dukha*).These people settled in the region when Tuva was annexed into the Soviet Union during World War II—although nearby areas may have been used by the Dukha for centuries [18]. Today, the Tsaatan are estimated to comprise a group of around 200 people, or about 30 families [19]. Most of these families occupy the Baruun Taiga area in the west, with a smaller number residing in the Zuun Taiga region along the eastern side of the Depression, outside the park’s protected area (Fig 1).The region is home to distinct wild reindeer populations. The animals exhibit markedly different behaviors, but occasionally crossbreed with domesticated stock (as do domestic bulls occasionally brought across the Russian border from Tuva).

**Fig 2:**
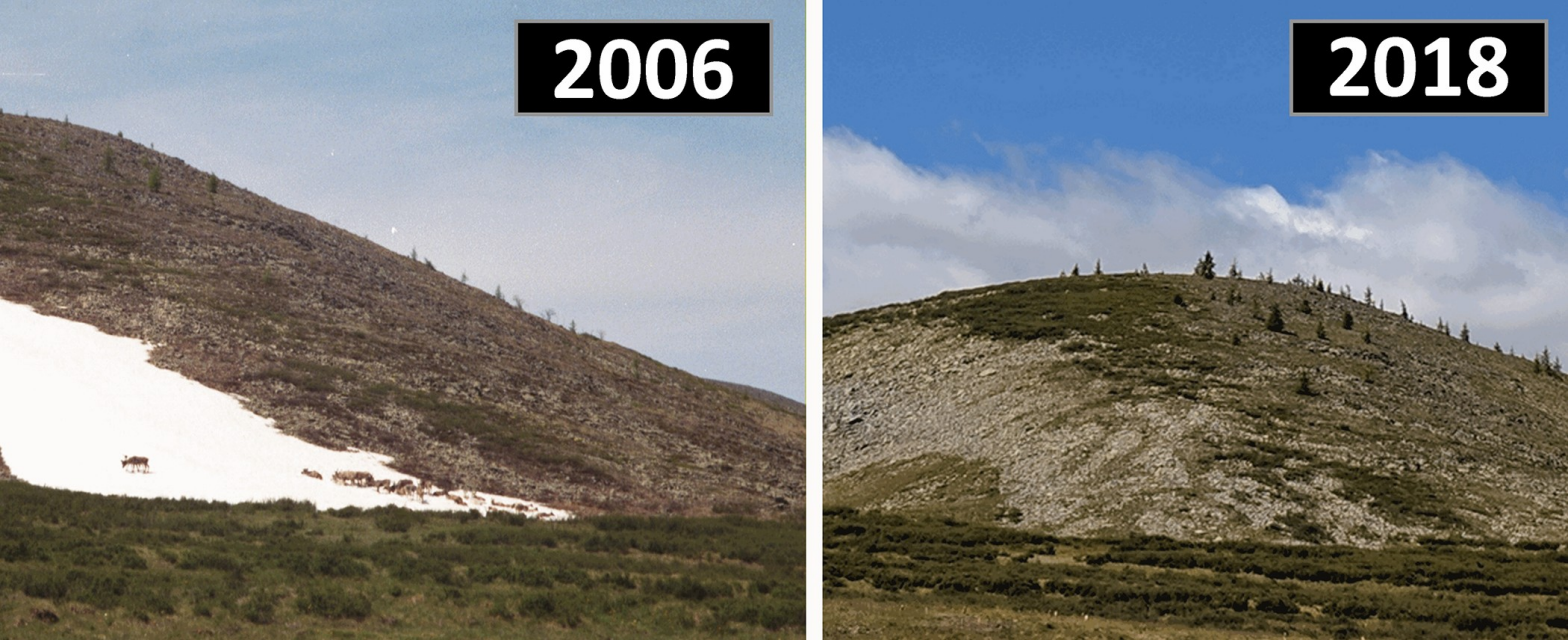
A (left); Image of a persistent snow and ice patch in Mengebulag taken in 2006, showing domestic reindeer using the patch, and B (right); the same patch in 2018, which local residents indicated had melted for the very first time.

**Fig 3:**
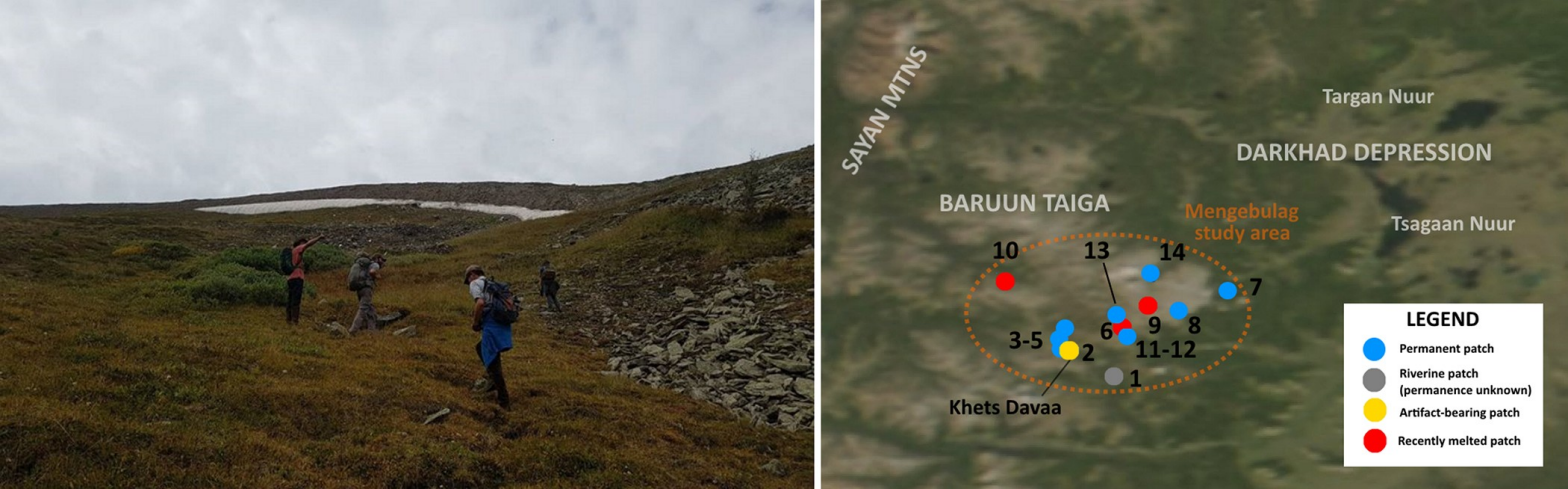
A, Left: Research team arriving at Ice Patch Three at Mengebulag, preparing to conduct mapping and survey. 3B, Right: Inset showing identified patch locations in relation to the study area and surrounding landscape. We acknowledge the use of imagery provided by services from NASA's Global Imagery Browse Services (GIBS), part of NASA's Earth Observing System Data and Information System (EOSDIS).

The study area, known as Mengebulag, a large tundra valley and popular summer pasture for Tsaatan reindeer herders located above 2000 masl within Baruun Taiga in the Sayan Mountains along the western lip of the Darkhad Depression. On a preliminary visit to the region in 2017, we identified ice patches between 1850–2580 masl in the vicinity of 51° N. Verbal discussions with residents indicated that during the summers of 2016–2018, previously permanent patches in Mengebulag melted for the very first time in collective memory (Fig 2). Recognizing the significance of this region to the understanding of reindeer pastoralism in Mongolia, and that organic cultural and biological materials contained within ice patches can decompose rapidly after their exposure[16], we selected this region for archaeological survey in the summer of 2018 (Fig 3).

During 2018 fieldwork at Mengebulag, we conducted systematic archaeological survey for material culture associated with pre-modern use of tundra zones in the Darkhad region. We also conducted informal interviews with eight local families living in summer pastures at Mengebulag, with the aim understanding A) the role ice patches play in contemporary reindeer herding, wild resource gathering, and subsistence; B) recent trends in ice melt extent and its impact on domestic reindeer; and C) ethnographic interpretation of organic artifacts recovered from ice patch localities in the Mengebulag area.

## Methods

### Ethnographic interviews

During informal ethnographic interviews with three families at their summer camps in the Mengebulag area (Fig 1), we asked informants questions pertaining to the location of perennial ice patches, the use of ice patches in reindeer pastoralism, and their interpretation of objects recovered from archaeological survey. Although we spoke with heads of households and elder individuals, the age range of participants was not documented. All participants provided verbal consent to the scientific use of their observations regarding ice patches in this study prior to interviews, which were conducted in Mongolian language and recorded using handwritten notes only. As discussions were anonymous, non-observational, and non-survey based (involving no recording of personal data), no institutional ethics review was necessary for this research, although all discussions were conducted in accordance with 2018 Guidelines and Rules of the Max Planck Society on a Responsible Approach to Freedom of Research and Research Risks.

### Ice patch archaeological survey

Archaeological research was conducted under research permit #18-6-1/2 (A/260) issued to the Northern Mongolia Archaeology Project and the National Museum of Mongolia from the Mongolian Ministry of Education, Culture, Science, and Sport on May 4^th^, 2018 –with authorization from the Baruun Taiga Special Protected Area (Ulaan Uul sum, Khuvsgul aimag, Mongolia). During late July and early August, 2018, we visited 11 ice patches in our study area identified through melt-season satellite imagery, along with two ice-free, former patch areas identified to us by ethnographic informants. Ice patch locations in the Mengebulag area were identified using 2016 and 2017 melt-season (July and August) satellite imagery from Google Earth and the NASA Earth Science Data Observatory. Ice patch permanence was assessed through discussion with area residents, who verbally assessed whether a given patch was perennial or melted during the summer., we conducted full-coverage horseback and pedestrian survey of the margins of 11 permanent ice patches at Mengebulag (Fig 3). Pedestrian surveys of ice patch areas were conducted at 1 meter transects radiating downslope from the current ice margin. In addition to artifact location, we documented ice patch aspect and maximum dimensions at the time of survey, although no detailed hydrologic or glaciologic data were collected. Find locations were documented using a handheld GPS at a precision of +/-3m. All specimens described in the research (Khets Davaa #1–5) are deposited in collections at the National Museum of Mongolia, Ulaanbaatar, Mongolia and available upon request. The individuals identified in Fig 3 have given written informed consent (as outlined in PLOS consent form) to publish this image.

### Radiocarbon dating

We sampled exterior wood shavings from organic artifacts for radiocarbon dating at the University of Oxford Radiocarbon Accelerator facility. We calibrated our measurements using the program CALIBomb (http://calib.org/CALIBomb/), using northern hemisphere Zone 1 from Hua et al [20] (and assuming a 0.5 year averaging among sampled tissue for the purposes of curve smoothing.

## Results

### Ethnographic interviews

**Fig 4:**
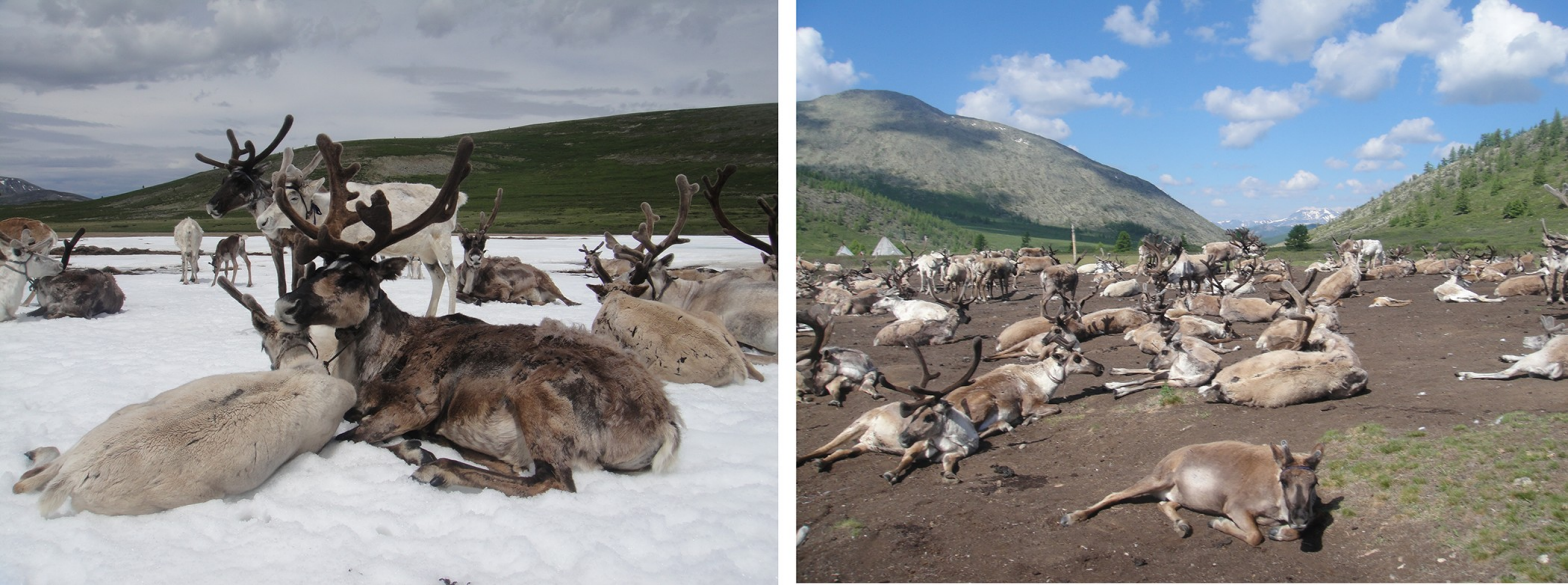
A (left); modern domestic reindeer cooling themselves on a riverbank snow patch in Mengebulag, and B(right); heat-stressed reindeer lying down in the dirt next to a summer camp at Sailag Gol, Zuun Taiga.

Discussions with residents of Mengebulag indicate that ice patches play a crucial role in reindeer pastoralism. Herders describe relying on the presence of ice patches for reindeer to escape summer heat stress and insects (Fig 4, left). As animal movements are limited by pasture availability, when these features melt, domestic reindeer will often search for other ice in the area, or seek heat relief by lying on the ground (Fig 4, right).

Informants also describe the importance of perennial ice patches as reliable fresh water sources. In our 2018 visit, only one summer camp was directly adjacent to a persistent ice patch. The families we visited described how many of the area’s patches had melted completely for the first time in their memory within the last three years (2016–2018). Informants suggest the ground may be drying out, and they point to the fact that while horses are commonly used near the Mengebulag camps today, just a few years ago this was impossible because of the swampy ground that was typical of the area. Many herders complained of declining pasture quality leading to bad health and even animal loss and death. When asked which criteria are most significant for pastoral use, one informant suggested that proximity to suitable camp locations and pasture was most important–and that these have been most severely affected by recent summer melting. On the basis of these interviews and observations, it appears that ice patch melting has a variety of negative outcomes for the viability of reindeer herding that may directly threaten this already vulnerable economic system (Table 1).

**Table 1:** Ethnographic uses of ice patches by reindeer herders in northern Mongolia, and the impacts of summer melting.

Ice patch use	Direct impact of summer melting	Indirect impacts
Reindeer cooling, resting place	Increased heat stress	Reduced herd health, lowered immune response
Reindeer insect relief	Increased insect activity	Increased parasite-borne disease
Drinking water (reindeer and human)	Reduced potable water availability	Landscape less sustainable for human and reindeer occupation

### Ice patch archaeological survey

**Fig 5:**
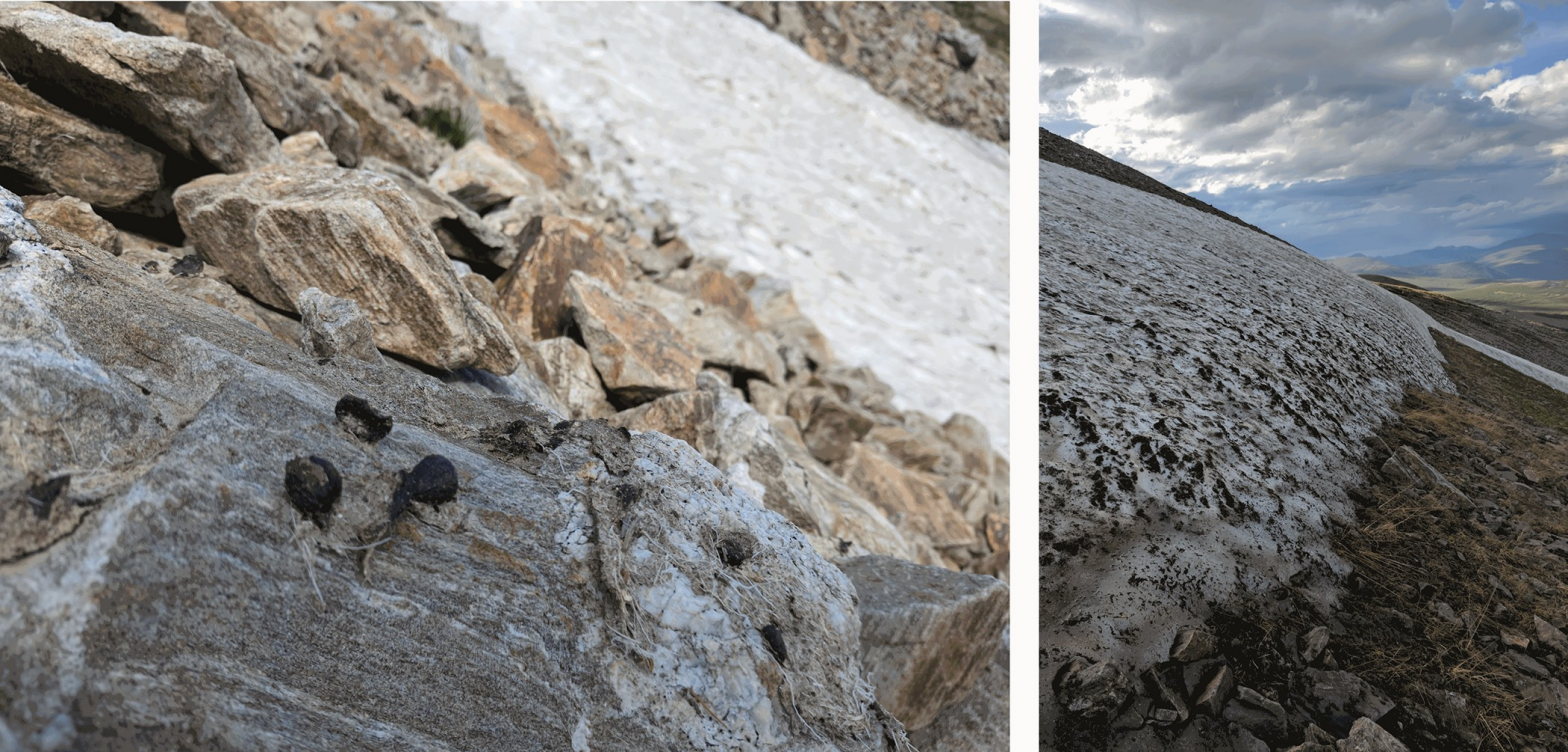
A (left); matted reindeer dung and hair recently melted out of an ice patch near summer camp at Mengebulag, and B(right); large ice patch with deposits of organic plant material.

At five of the patches studied using archaeological survey, we recovered plant and animal materials, presumably non-cultural in origin, emerging directly from the melting ice. Although not directly related to human activity, these finds demonstrate the presence of interpretable paleoecological material (Fig 5, right) is preserved in the Mongolian ice patches. One of these patches, Patch Five, was located directly adjacent to a Tsaatan summer camp, and showed extensive evidence of exploitation by domestic reindeer–including tracks, feces, and large mats of reindeer hair (Fig 5, left). While we did not date the ecological material melting out from this patch, it appears to be melting rapidly and likely contains organic material related to reindeer activity spanning the historic occupation of this region and perhaps earlier.

### Archaeological discoveries

**Fig 6:**
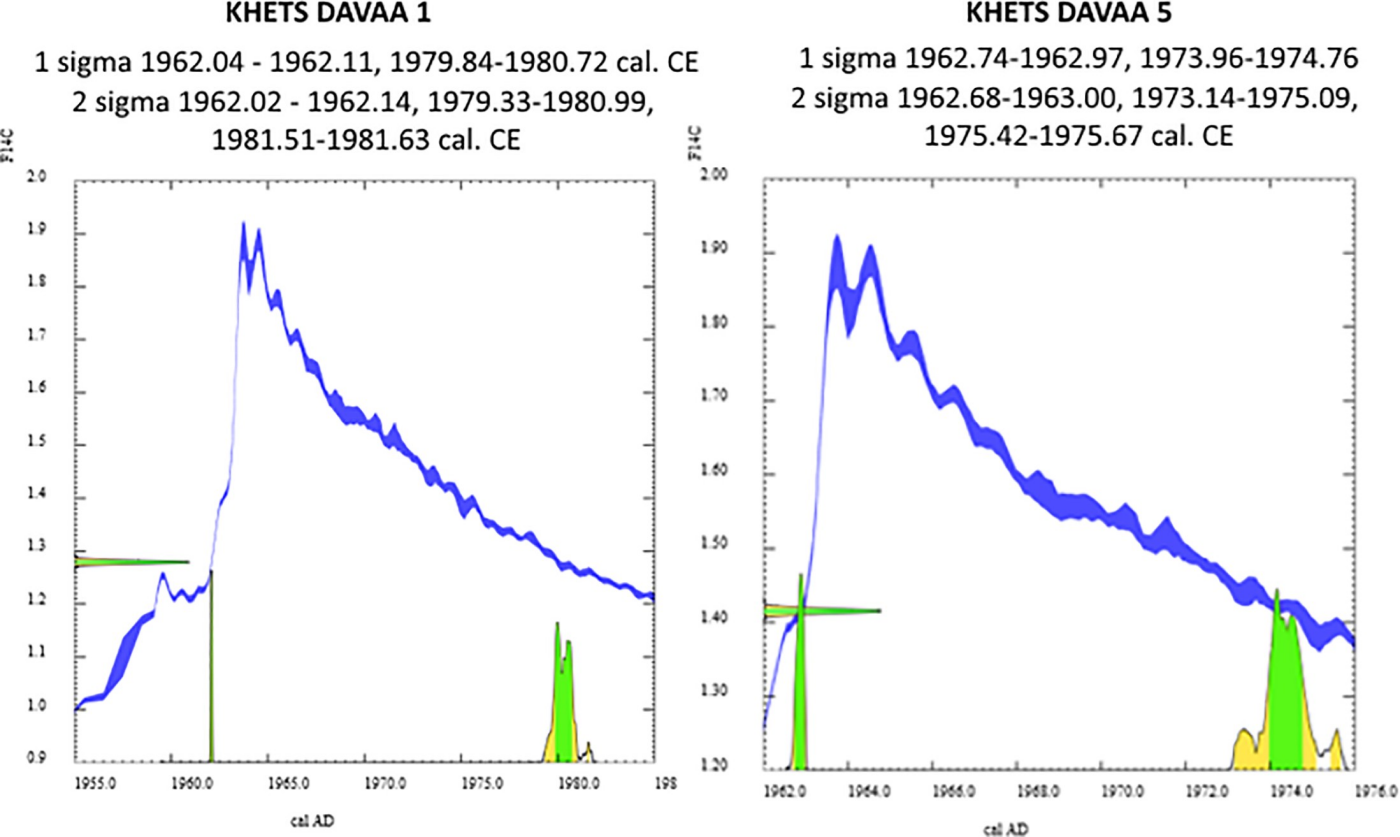
Calibration curve intersection for artifacts from Khets Davaa #1 (left) and #5(right), calibrated using NH1 curve, 0.5 yr smoothing, using Calibomb Software (http://calib.org/CALIBomb/). If the two pieces originate from a single piece of wood, they most likely relate to the early 1960’s. Alternatively, they may have been deposited either separately or together during the late 1970’s or early 1980’s.

At the site of Khets Davaa, we discovered a number of organic wooden artifacts exhibiting cultural modification from an ice-free patch location that (according to informants) melted completely for the first time in 2016–17. Although the site is today unwooded and above the tree line, we recovered several pieces of cylindrical, unmodified wood that appear to be “manuports” brought to the site through human activity. In addition, we recovered three artifacts exhibiting cultural modification. The first of these is a stick ~2.5 cm in diameter, exhibiting cut marks along the bottom margin, probably produced by a metal hatchet (Fig 6). One possible function for such an object might be as a ‘scaring stick.’ Similar objects–worked branches placed upright into the snow–have been used to direct the movements of wild reindeer during hunting [21]. Given the site’s location atop a key travel corridor and mountain pass and the presence of wild reindeer, this explanation seems plausible, although other factors must also be considered (for example, in snow science, vertical sticks placed in snow may be used to measure snow depth).

**Fig 7:**
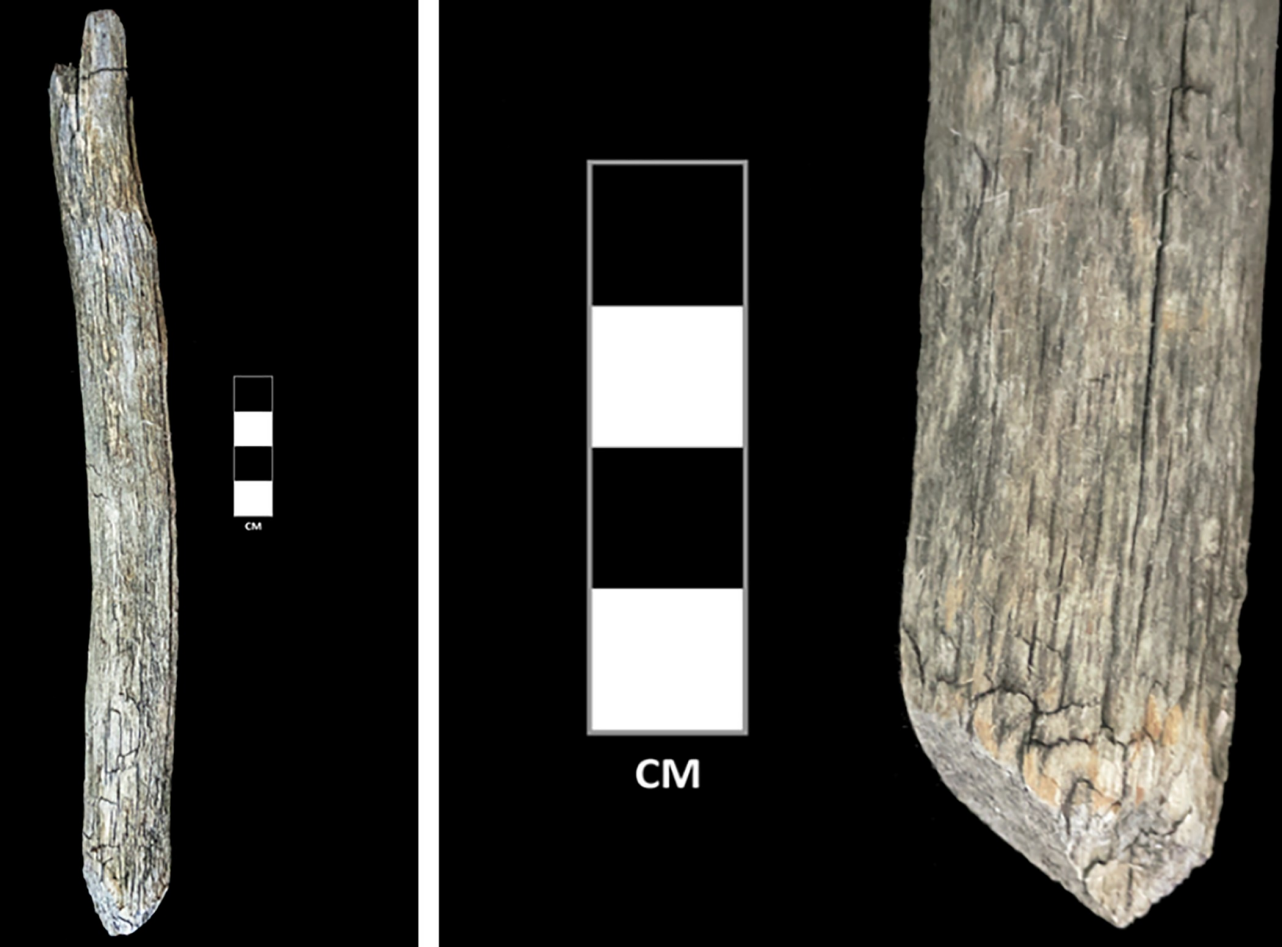
Modified wooden branch artifact from Khets Davaa, with closeup on modified edge.

**Fig 8:**
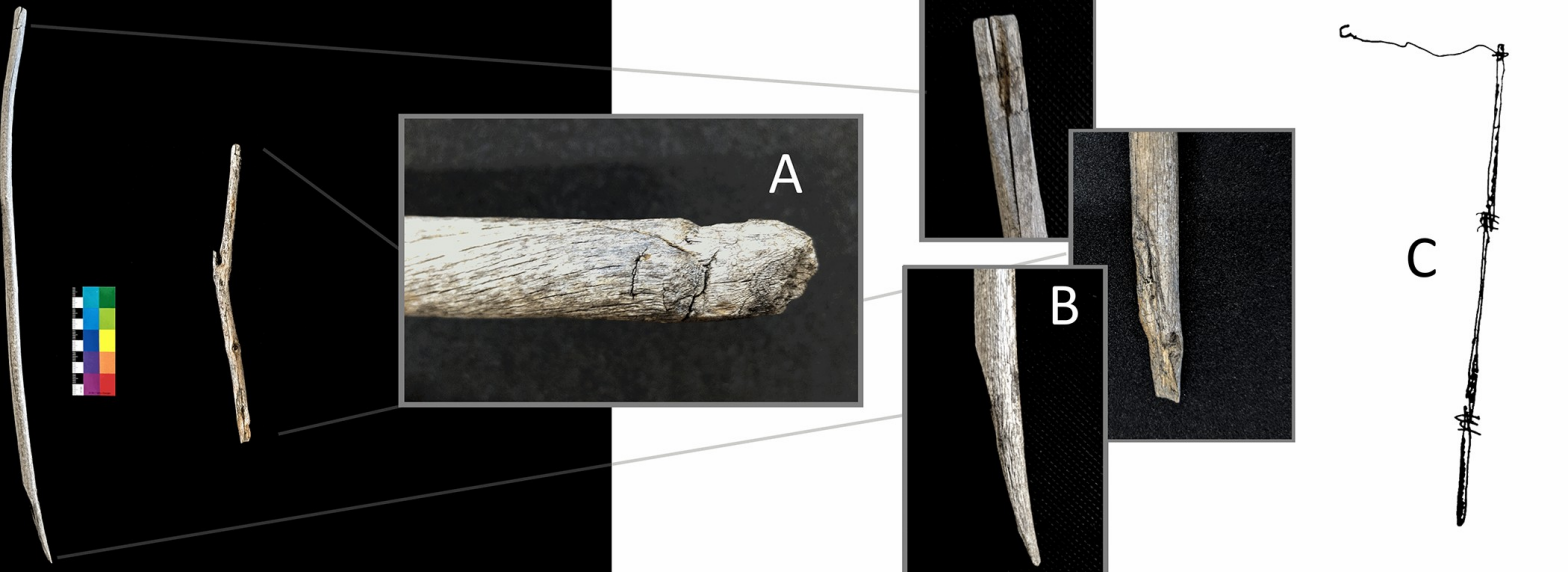
Modified willow branch artifacts recovered from Khets Davaa in Baruun Taiga, northern Mongolia. Center inset (A) shows nocked/carved end of the artifact, presumably to facilitate tying. The other ends of both pieces have been beveled and “scarfed” to facilitate lashing to similar pieces (B), perhaps as part of a fishing pole (C)—as in this sketch drawn from ethnographic interviews (artist: Bayandalai).

**Fig 9:**
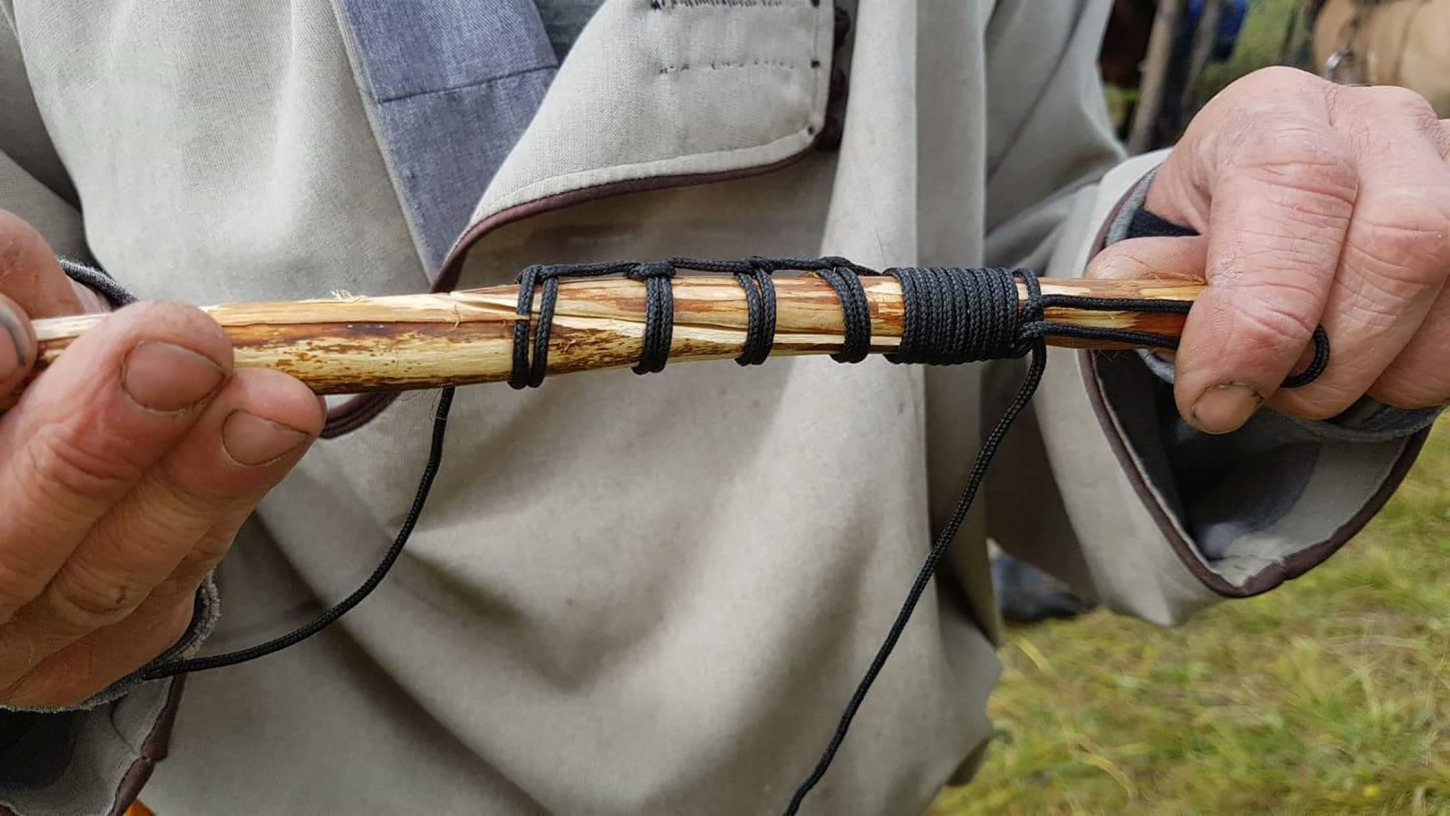
Ethnoarchaeological recreation based on ethnographic informant’s description of traditional technique for lashing willow branches, used in the production of fishing poles.

Our second discovery consists of two worked willow branch artifacts that may have originally been a single object. The two pieces are each ~1.5cm in diameter. The first is a smooth, worked shaft around a half-meter in length. Both sides of the artifact have beveled, intentionally worked edges that are offset from each other by roughly 90 degrees (Fig 7, left). At least one of these two bevels appears to have been intentionally “scarfed”–that is, produced with a bevel as well as a lip, in order to produce a secure joint when lashed to a similar, opposing edge (Fig 8). Discussions with elder ethnographic informants revealed that this technique of edge preparation was used traditionally by Tsaatan people in the 20^th^ century, as a means of producing a strong yet flexible joint (Fig 9). The second willow branch consists of a much shorter piece with one, similarly scarfed terminus, opposite a notched edge that appears to have been used to secure a tie or lash (Fig 7, second from left). Because these objects were recovered in close proximity, it is possible that they originally belonged to the same object, and were subsequently separated. Two separate ethnographic informants suggested to us that this pair of objects was likely part of a composite fishing pole (Fig 7, right), with the notched end serving as the tie for the fishing line. The lashed willow branches would have provided a rigid, yet flexible pole.

Radiocarbon assays on these two specimens both yielded “modern” ages (Table 2). Their intercept with the calibration curve suggests a deposition event during the late 1960’s or late 1970’s (Khets Davaa 1: 1962, 1979–1981, 2 sigma calibrated range; Khets Davaa 5, 1962–3, 1973–1975, 2 sigma calibrated range; Fig 6). The relatively recent dates strengthen the relevance of the ethnographic identification of this object as a fishing pole. However, it should also be noted that a similar technique is used to join short lengths of bone and wood across the circumpolar north, and absent further information, these pieces could have been part of a number of different types of object. Because organic artifacts degrade quickly once free of the preservative ice, our findings suggest that contemporary levels of ice melt exceed any during recent decades—perhaps going back a half century or more.

**Table 2:** Radiocarbon dates from artifacts recovered at Khets Davaa, near Mengebulag in Ulaan Taiga Special Protected Area. 14C results for modern values are presented as proportion of modern carbon.

Sample	Material	14C measurement *(fraction modern carbon)*	+/-	Lab number	Calibrated date range (2 sigma)
Khets Davaa #1	Willow branch	1.27911	0.00312	OA38062	1962.02–1981.63 cal. CE
Khets Davaa #5	Willow branch	1.41541	0.00330	OA38061	1962.68–1975.67 cal. CE

## Discussion

Results from these preliminary ethnographic and archaeological investigations of persistent snow and ice patches in the high mountain zones of northern Mongolia indicate that these locations play a crucial role in domestic reindeer pastoralism, which is directly threatened by summer melting resulting from climate change. Ethnographic informants describe reindeer using *munkh mus* for drinking water, regulating body temperatures, and for relieving predation by insects. In some cases, recent melting has resulted in the complete loss of these historically permanent landscape features.

The absence of ice patches curtails the animals’ ability to thermoregulate, and their inability to escape insects may also increase exposure to parasites and vector-borne disease (Laaksonen et al. 2010). Such behaviors are also noted among the semi-domesticated reindeer of the Saami people in northern Scandinavia and wild caribou of North America [14,22–26]. In many of these cases, herds are adapting to the disappearance of ice patches by moving to higher elevations or more northern latitudes [23,27]. However, this adaptation is not available to the Tsaatan reindeer herders, as they already occupy the highest available zones within Mongolia, and are hemmed in to the north and west by the international border with Russia.

Access to fresh water for humans reindeer is also an important function provided by ice patches that is threatened by summer warming. Several informants described proximity to suitable pasture and camp locations as the most important factor in determining patch use for pastoral purposes, and indicated that those closest to camp were subjected to the most severe recent melting (e.g. Fig 2). Pastorally-utilized ice patches in the study area the lowest elevation among identified patches. While there is still enough drinking water for humans and stock today, informants complained of drying pastures. If the melting of ancient ice, and the trend toward increasingly drier conditions continues, it may have a disastrous impact on reindeer, as they are especially sensitive to water availability [9,28].

The use of new pastures in the Darkhad region is also prohibited by regulations governing the area’s “strictly protected” conservation zones. Despite receiving financial state support for increasing their herds, with these restrictions in place, current pastures suffer increasing degradation due to larger herd sizes. In 2018 conversations, informants mentioned that lichen and plants eaten by reindeer typically need three years to fully recover—meaning that restricted mobility has dire consequences for pastured degradation. Under these paired socioeconomic and ecological circumstances, ice patches appear even more important for the Tsaatan and their reindeer. Other patch qualities, beyond those explicitly mentioned by informants (such as having a gentle, accessible slope or limited snow/ice depth) may also be important, as well as influenced by the melting process. As continued warming is anticipated in the future, we expect that ongoing melting of ice patches will cause new sources of stress and disease for livestock (such as harassment from oestrid flies, heat stress, and mosquito-borne parasites), reducing the viability of summer pastures threatening the sustainability of reindeer pastoralism.

Our archaeological survey provides support for the idea that ice patches contain key insights into the role of mountain zones in the poorly understood history of domestic reindeer in Mongolia. Our investigation identified one patch that showed extensive use by modern reindeer, as evidenced by a large amount of previously frozen dung, hair, and other organic material. Future investigation of this patch and others yielding plant and animal material during peak summer melt, paired with radiocarbon dating and more nuanced paleoecological analysis, may provide important new data necessary to reconstruct the antiquity of domestic reindeer use in northern Mongolia.

Moreover, the discovery of at least two artifacts shows that taiga environments were exploited by Tsaatan people for acquiring wild resources–possibly fish–after their arrival to the region in the mid-20th century. The radiocarbon dates suggest the most likely period for the deposition of the artifacts was the early 1960’s during the Soviet-era collectivization period, or in the late 1970’s. Ethnographers have documented the former interval as a particularly harsh transition for the Dukha people, and a period during which traditional hunting practices were largely banned, and reindeer herding was not supported under state-run livestock policies [18]. During this time most young people in the region moved to the county seat of Tsagaan Nuur to work on the newly-established collective fishery [18].

Ice patch melting in the region is part of a larger global phenomenon [29]. Our results indicate that northern Mongolia may have experienced historically unprecedented ice patch melting over recent decades. The extreme melting observed during the 2018 season, and the degraded condition of the exposed artifacts that were recovered at Khets Davaa, indicates that summer warming is an urgent threat to the material culture preserved in the ice patches in Darkhad and other subarctic regions of northeast and Central Asia.

## Conclusion

While the emergence of pastoral economies in Central Asia was one of the most important economic transitions in human history, many of the dynamics underlying this transition are poorly understood. This is particularly true of the archaeological record of high-mountain zones. Strong circumstantial evidence suggests that these areas may have been crucial to the early development of herding lifeways; however, due to their environmental and depositional characteristics, these areas retain little direct evidence of how they were used in the deep past. In Mongolia, mountain zones host unique species of domestic animals (reindeer and yak), that provide the economic foundation of some modern lifeways, but evidence of the process by which they were domesticated is almost absent from the archaeological record.

Our investigations in northern Mongolia indicate that persistent snow and ice patches–long known to be important loci for reindeer hunting–play a central role in modern reindeer pastoralism. The degradation and loss of these features in summer pastures threaten the viability of this herding lifestyle. Archaeological surveys and ethnographic research of ice patches in one important tundra zone in Mongolia’s Darkhad Basin provide the first direct material evidence of exploitation of this region in the mid-twentieth century, and suggest that contemporary melting exceeds levels seen in at least half a century. Culturally modified wood recovered from a melting ice patch suggest use of the area for summer pastures and travel corridors in modern times (as well as for wild resource gathering such as fishing) in the mid-20th Century, after documented migration to the region from southern Russia. While future research may yield important cultural and paleoenvironmental material relevant to understanding the deeper chronology of reindeer domestication and pastoral use of high altitudes, ice patches and the material they contain are urgently threatened by warming temperature and climate change.
